# MiTree: A Unified Web Cloud Analytic Platform for User-Friendly and Interpretable Microbiome Data Mining Using Tree-Based Methods

**DOI:** 10.3390/microorganisms11112816

**Published:** 2023-11-20

**Authors:** Jihun Kim, Hyunwook Koh

**Affiliations:** Department of Applied Mathematics and Statistics, The State University of New York, Korea (SUNY Korea), Incheon 21985, Republic of Korea

**Keywords:** microbiome data analysis, web cloud computing, human microbiome, machine learning, tree-based methods, classification and regression

## Abstract

The advent of next-generation sequencing has greatly accelerated the field of human microbiome studies. Currently, investigators are seeking, struggling and competing to find new ways to diagnose, treat and prevent human diseases through the human microbiome. Machine learning is a promising approach to help such an effort, especially due to the high complexity of microbiome data. However, many of the current machine learning algorithms are in a “black box”, i.e., they are difficult to understand and interpret. In addition, clinicians, public health practitioners and biologists are not usually skilled at computer programming, and they do not always have high-end computing devices. Thus, in this study, we introduce a unified web cloud analytic platform, named MiTree, for user-friendly and interpretable microbiome data mining. MiTree employs tree-based learning methods, including decision tree, random forest and gradient boosting, that are well understood and suited to human microbiome studies. We also stress that MiTree can address both classification and regression problems through covariate-adjusted or unadjusted analysis. MiTree should serve as an easy-to-use and interpretable data mining tool for microbiome-based disease prediction modeling, and should provide new insights into microbiome-based diagnostics, treatment and prevention. MiTree is an open-source software that is available on our web server.

## 1. Introduction

The human microbiome is the aggregate of all microbes that reside on and inside different organs (e.g., intestines, oral cavities, nasal cavities, dermis, respiratory apparatus, genitals, etc.) of the human body. Resident microbes play crucial roles in their human host’s health or disease through the channels of immunologic or metabolic regulations, digestive processes, synthesizing vitamins, preventing bacterial colonization and so forth [1,2,3]. The advent of next-generation sequencing has greatly advanced the field of human microbiome studies, while improving the accuracy of microbiome quantification at a substantially lowered price. Currently, investigators in academia and industry are seeking, struggling and competing to find new ways to diagnose, treat and prevent human diseases through the human microbiome [4,5,6,7,8,9,10,11].

However, one of the most challenging issues in human microbiome studies is in the high complexity of microbiome data [12,13,14]. The abnormal characteristics of microbiome data can be described as follows: First, it is high-dimensional data including various microbial taxa at different taxonomic ranks (e.g., phyla, classes, orders, families, genera and species). Most of the taxa are also rare in abundance including excessive zeros. The abundance distribution can also be highly irregular and overdispersed across units (i.e., study subjects or individuals), indicating that some people can be rich in some taxa while other people are surely poor in them. The microbial taxa can also be functionally or phylogenetically related to each other. To handle such highly complex microbiome data, machine learning can be a useful and promising approach.

However, many of the current machine learning algorithms are in a “black box”, that is, they are difficult to understand and interpret. For instance, they do not care about the exact forms of the relationships between microbial taxa and their host’s health or disease status as long as they can make high-accuracy predictions on the output (i.e., a host’s health or disease status) using inputs (i.e., microbiome); however, we are interested in more than just this information. For instance, we would like to figure out which microbial taxa are culprits or fellow travelers and how they are related to human diseases. Machine learning also requires professional programming skills and high-end computing devices. In addition, many of the clinicians, public health practitioners and biologists in the microbiome field are not professional programmers, and they do not always have fancy computers. To summarize all the above issues, it is difficult and very demanding to handle highly complex microbiome data through machine learning for clinicians, public health practitioners and biologists who are curious about many different aspects in microbiome-based disease prediction. 

In this paper, we introduce a unified web cloud analytic platform, named MiTree, for user-friendly and interpretable microbiome data mining. MiTree employs tree-based learning methods, including decision tree [15], random forest [16] and gradient boosting [17,18], which are well understood and suited to human microbiome studies. The tree-based methods split the input (microbiome) space into a number of partitions for different units in a non-parametric way and make a prediction on the output (health or disease status) for each partition; and therefore, they are more robust to possible discrete or irregular patterns of the relationships than the linear model-based methods (e.g., ridge [19], lasso [20] and elastic net [21]). In other words, as we described, most of the taxa are rare and have zero-inflated features, and the abundance distribution can also be highly irregular and overdispersed across units. In turn, this can lead to highly discrete or irregular patterns of the relationships toward a host’s health or disease status, to which the splitting rules of algorithmic tree-based methods are better suited than the linear model-based methods that make prediction lines [22,23,24,25]. Decision tree [15] is the simplest form based on a single tree, while random forest [16] and gradient boosting [17,18] combine a sheer number of trees. While it depends on the true underlying relationships, analytic schemes, the nature of study data and so forth, it is common that decision tree [15] is the easiest to interpret, the fastest in computation, but the least accurate in prediction, while random forest [16] and gradient boosting [17,18] are more accurate in prediction but at the expense of a little less interpretability and even heavier computation (Table 1). 

However, we note that the gain in prediction accuracy using random forest [16] or gradient boosting [17,18] instead of decision tree [15] is substantially greater than the loss in interpretability (Table 1). It is also common that investigators gladly tolerate some computational time as long as they can obtain better results. Moreover, since MiTree runs gradient boosting [17] (which is usually regarded as the slowest learning method) using the software package, XGBoost 1.7.5.1 [18], it enables fast C++ implementations and it is also computationally manageable. Therefore, we suggest using random forest [16] or gradient boosting [17,18] as the main analytic method, while decision tree [15] can be used just for reference. We describe and discuss random forest [16] and gradient boosting [17,18] further, in detail, in a later section, Materials and Methods: Random Forest vs. Gradient Boosting. 

Our prior web cloud platforms, i.e., MiCloud [26], MiPair [27], MiSurv [28] and MiMed [29], have mainly focused on significance testing using model-based methods. MiTree is well distinguished from them as a data mining tool for microbiome-based disease prediction using algorithmic tree-based methods. Furthermore, covariate-adjusted analyses are necessary to properly control for potential confounders (e.g., age and sex), especially for observational studies, yet there is no other web cloud platform that can handle covariate-adjusted analyses in microbiome-based disease prediction [30,31,32,33,34,35,36,37,38]. We emphasize, here, that MiTree can handle both classification and regression problems through a covariate-adjusted or an unadjusted analysis, and as such, MiTree can apply to cross-sectional studies of randomized controlled trials or observational studies with a binary or continuous output variable (Table 2). The results from MiTree are also easy to understand and interpret with good visualizations for important disease predictors and their delicate relationship patterns with the host’s health or disease status. It is also engaging that, as in MiMed [29], MiTree employs ChatGPT to help users to easily search for the microbial taxa that are found as important disease predictors. There are numerous microbial taxa at different taxonomic ranks, and it is not easy to catch or distinguish their names. Thus, we need help from a well-trained AI language model to find prior knowledge. This plugin facility of ChatGPT can be useful for verification purposes to see if they have been reproduced or newly discovered, while further enhancing the user-friendly operation of MiTree. Overall, MiTree should serve as an easy-to-use and interpretable data mining tool for microbiome-based disease prediction modeling and should provide new insights into microbiome-based diagnostics, treatment and prevention.

## 2. Materials and Methods

### 2.1. Random Forest vs. Gradient Boosting

Random forest [16] is a bootstrap aggregation method that averages predicted outputs (health or disease status) in an ensemble of bagged trees that are created using randomly selected inputs (taxa). The process of randomly selecting inputs (taxa) is well suited to human microbiome studies, while decorrelating microbial taxa that tend to be functionally or phylogenetically related and reducing the variability of predicted outputs (health or disease status) through averaging. It can also robustly adapt to varying sparsity levels in a high-dimensional setting, in which a high (low) sparsity level represents the situation in which only a few taxa (many taxa) are related to the host’s health or disease status. 

As for random forest [16], gradient boosting [17,18] also combines a number of trees, but the trees are grown (boosted) sequentially through weak learners that make a strong committee in the end. The weak learners are simple decision trees [15] (e.g., a stump with two terminal nodes) that are slightly better than random prediction. Gradient boosting [17,18] updates its predicted outputs (health or disease status) very slowly applying a small learning rate to the ones modified by weak learners iteratively. Then, the predicted outputs (health or disease status) are fine-tuned for highly delicate partitions of the input (microbiome) space. Gradient boosting [17,18] has been considered to be one of the most precise machine learning approaches [22], and importantly, it allows any differentiable loss functions to be considered. 

While random forest [16] and gradient boosting [17,18] bear some resemblance to each other, they are also well distinguished from each other. We can not make any easy judgement on which method is better. One method is not superior to the other in all situations and contexts, although we would claim that both are highly reasonable, robust, and accurate methods that are well suited to human microbiome studies. 

### 2.2. Training Processes

The underlying training processes to find the optimal tuning parameters for decision tree, random forest and gradient boosting are as follows. First, for decision tree [15], MiTree takes a top-down greedy approach known as recursive binary splitting to find the groups of units and to estimate their predicted values (i.e., their average output values) while minimizing training errors. However, the resulting tree can be huge in size with many leaves (i.e., high complexity), and thus can overfit the data. Hence, MiTree applies a tree pruning approach known as cost-complexity pruning to find the optimal tree size (i.e., the optimal number of leaves) through cross-validation. Second, for random forest [16], MiTree aggregates multiple trees (the default is 5000 trees), and there is no overfitting issue with an increase in the number of trees. For random forest, the only tuning parameter is the number of randomly selected taxa. MiTree finds the optimal number of randomly selected taxa through cross-validation. Finally, for gradient boosting [17,18], MiTree grows the tree slowly using a small learning rate (the default is 0.005) possibly with the regularization using $L_{2}$ penalty [18]. However, gradient boosting can overfit the data as the number of trees (i.e., the number of iterations) increases. Hence, MiTree finds the optimal number of trees, as well as the optimal number of splits in each tree known as the interaction depth, in a grid search through cross-validation. More details on the available loss functions, cross-validation settings and other model specifications can be found in the following Section 3.3, Section 3.4 and Section 3.5.

### 2.3. R Libraries

MiTree 1.0.1 is written in R language. The underlying R libraries are as follows: For the decision tree, we used ”rpart” for training and validation and ”rpart.plot” for visualization. For random forest, we used ”randomForest” for training and validation and ”edarf” for visualization. For gradient boosting, we used ”xgboost” for training and validation and ”SHAPforxgboost” for visualization. For the user interfaces and server functions, we used ”shiny”. 

### 2.4. Web Server and GitHub Repository

MiTree is an open-source software available on our web server (http://mitree.micloud.kr, 10 October 2023). We deployed our web server using ShinyProxy 2.6.1 (https://www.shinyproxy.io, 10 October 2023) and Apache2 (https://httpd.apache.org, 10 October 2023) on Ubuntu 20.04 (https://ubuntu.com, 10 October 2023). Our web server runs on a computing device with an Intel Core i9-12900 (16-core) processor (Intel, Santa Clara, CA, USA) and 64 GB DDR4 memory (Samsung, Seoul, Korea), and accepts up to ten concurrent users. When our server is busy, users can also run MiTree using their local computer through our GitHub repository (https://github.com/jkim209/MiTreeGit, 10 October 2023). We are devoted to mainlining our web server and GitHub repository at the highest quality. 

### 2.5. Data Availability

The data we used in this study are public subgingival microbiome data for non-e-cigarette users at a baseline visit [39], which were sequenced using 16S rRNA amplicon sequencing [40,41] and quantified using QIIME2 6.0 [37] based on an expanded human oral microbiome database (eHOMD) [42]. The raw sequence data are publicly available at the repository of the NCBI Gene Expression Omnibus (http://www.ncbi.nlm.nih.gov/geo, 10 October 2023) with access number GSE201949. We also uploaded the processed data as example data (see *Data Input: Example Data* on our web server: http://mitree.micloud.kr, 10 October 2023).

### 2.6. Code Availability

MiTree is an open-source software available on our web server (http://mitree.micloud.kr, 10 October 2023) or runs on a local computer through our GitHub repository (https://github.com/jkim209/, 10 October 2023 MiTreeGit). All the source codes and software manuals can also be found there. 

## 3. Results

This section is devoted to describing all of MiTree’s data processing and analytic modules. We demonstrate each module with diagnostic and clinical interpretations through an example oral microbiome study to predict gingival inflammation (output) using microbial taxa (inputs) [39] (see the subsection, named *Example*). The original study has already been published in [39], and the raw sequence data are publicly available (see a later section, named Section 2.5). The data we used are the subgingival microbiome data for non-e-cigarette users at the baseline visit [39]; the data were sequenced using 16S rRNA amplicon sequencing [40,41] and quantified using QIIME2 [37] based on the expanded human oral microbiome database (eHOMD) [42]. To help our users better understand and follow each module of MiTree, we also uploaded the processed data as example data (see *Data Input: Example Data* on our web server: http://mitree.micloud.kr, 10 October 2023). 

### 3.1. Data Processing: Data Input, Quality Control and Data Transformation

In the *Data Input* module, for MiTree, it is necessary to upload three data components: (1) a feature table (i.e., a count table for the features, operational taxonomic units (OTUs) or amplicon sequence variants (ASVs)), (2) a taxonomic table (i.e., for taxonomic allocations at seven taxonomic ranks, kingdom, phylum, class, order, family, genus and species), and (3) metadata (i.e., for unit information on health/disease output, demographics, etc.). Users can upload the components using an integrative format, called phyloseq [43] or using individual files. 

Then, in the *Quality Control* module, users need to select (1) a kingdom of interest (default is Bacteria), (2) a minimum library size (i.e., total read count) for the units to be kept in downstream analysis (default is 3000), (3) a minimum mean proportion for the features (OTUs or ASVs) to be kept in downstream analysis (default is 0.002%), and (4) erroneous taxonomic names to be removed in the taxonomic table. MiTree displays the sample size and the numbers of features (OTUs or ASVs), phyla, classes, orders, families, genera and species using summary boxes. MiTree also visualizes the library sizes across units and the mean proportions across features using interactive histograms and box plots. 

Finally, in the *Data Transformation* module, users can transform the data into the following four widely used data formats: (1) centered log ratio (CLR) [44] (default), (2) rarefied count [45], (3) proportion, and (4) arcsine root. Among those, CLR [44] is the most widely used data format in human microbiome studies to normalize the data and to mitigate the compositional constraint, and the CLR transformed data are in a continuous scale. The rarefaction [45] is to fix the library sizes across units while maintaining the nature of the data as counts. The proportion is to fix the library sizes across units to be one (i.e., 100%), but the nature of the data is transformed from counts to compositions. Finally, the arcsine root stabilizes the variances in proportion across units, and the arcsine-root-transformed data are in a continuous scale. For reference, users can download all the transformed data. 

Example: We uploaded the example oral microbiome data, performed quality controls using default settings, and then transformed the data into four different data formats (Figure S1). 

### 3.2. Data Mining

The *Data Mining* module is to conduct microbiome data mining using the tree-based methods, decision tree [15], random forest [16] and gradient boosting [17,18] for each taxonomic rank. All these methods can address both classification (in which the health or disease output variable is binary) and regression (in which the health or disease output variable is continuous) problems through covariate-adjusted or unadjusted analysis. For the covariate-adjusted analysis, first, MiTree fits a generalized linear model (i.e., the logistic regression for classification and the linear regression model for regression) including only the health or disease status as the output variable and the covariates (e.g., age, sex) as the input variables (excluding the microbiome) using maximum likelihood estimation, and then obtains residuals. The residuals are the remaining portion of the output (e.g., health or disease status) after the covariates are explained (e.g., age and sex). The conventional parametric approach is also reasonable in this step because the output (e.g., health or disease status) and inputs (e.g., age, sex) are typical variables and the complex microbiome portion has not been involved. Then, in the following analysis using a tree-based method, the residuals are used as the output variable and the microbial taxa are used as input variables [46]. Note that once users select to conduct the covariate-adjusted analysis, both classification and regression problems are treated as a regression problem in the end because the residuals from the logistic regression (i.e., Pearson’s residuals) are in a continuous scale. 

Importantly, a the beginning, MiTree splits the data into two non-overlapping portions, i.e., test data (20%) and training data (80%), and reports the test errors for the comparison in prediction accuracy across the three tree-based methods, decision tree [15], random forest [16] and gradient boosting [17,18]. Then, users can choose a method with the lowest test error for the highest prediction accuracy.

Ask ChatGPT: The plugin facility for ChatGPT is available at the end of each analytic module (*Data Mining: Decision Tree*, *Data Mining: Random Forest*, and *Data Mining: Gradient Boosting*, respectively). Users can ask a query “Tell me about the roles of (a microbial taxon) on (a human disease)”, that is, each analytic module reports microbial taxa that are found as important disease predictors. Then, users can select a microbial taxon among those important disease predictors and search for prior studies on its roles on the output (e.g., health or disease status) variable. As in MiMed [29], this module also returns the search results from Google Scholar and PubMed for better re-verification purposes. To avoid duplicate explanations, we demonstrate its use at the end of Section 3.5 only. 

### 3.3. Data Mining: Decision Tree

In this module, users can conduct microbiome data mining using decision tree for both classification and regression problems through recursive binary splitting and cost-complexity pruning [15]. First, users need to select an output (e.g., health or disease status) variable. Then, users select a data format among CLR (default), rarefied count, proportion and arcsine root. Then, users select covariates (e.g., age and sex) for the covariate-adjusted analysis or not for the unadjusted analysis. Then, users select a loss function, cross entropy (default) or Gini impurity for classification and mean squared error (default) for regression. Then, users select (i) leave-one-out cross-validation (LOOCV) (default), 5- or 10-fold CV, (ii) the minimum number of units in a node to for a split to be an attempt (the default is 10), and (iii) the minimum number of units to be included in each leaf (the default is 5). Finally, users select the taxonomic ranks to be surveyed ”from phylum to genus” (default), which is for 16S data [40,41], or ”from phylum to species”, which is for shotgun metagenomic data [47]. MiTree reports the main results using a top-down tree structure and a table for the number of units that belong to each leaf, the predicted output value for each leaf minus the overall predicted output value for directional interpretation on if the units in each leaf make smaller (−) or larger (+) output values than the overall average output value. MiTree also reports supplemental results using a CV error plot to show the underlying CV process for cost-complexity pruning to search for an optimal tuning parameter (the number of leaves or complexity parameter) value.

**Figure 1:**
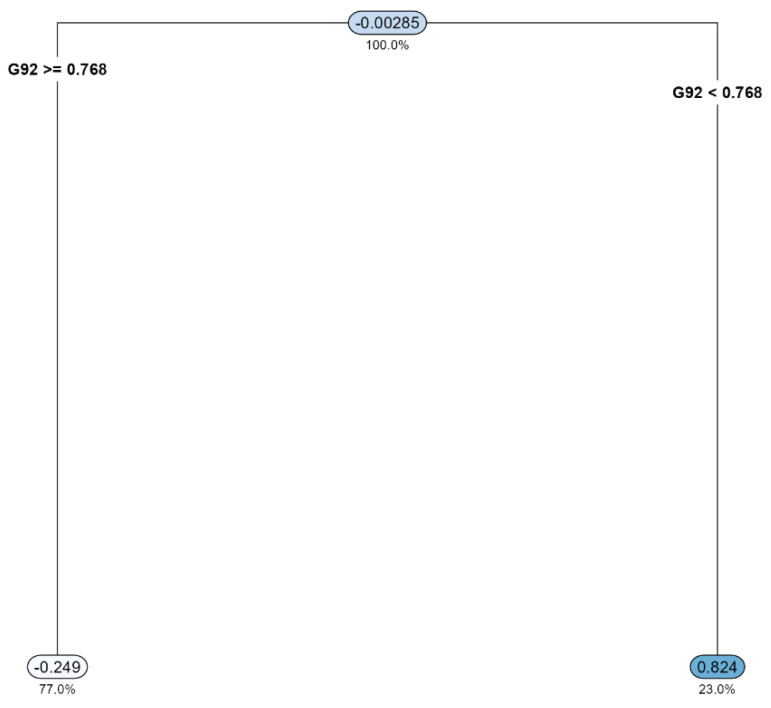
The top-down tree structure resulting from decision tree. G92: *Arachnia*.

Example: We selected gingival inflammation as the output variable and CLR [44] as the data format. Then, for covariate-adjusted analysis we selected age, sex and the frequency of brushing teeth as covariates. Then, we used all the default settings for the rest of the widgets. In summary, based on the results, we found, at the genus level, that *Arachnia* was an important predictor for gingival inflammation (Figure 1). We also estimated, in a diagnostics sense, that 77.0% of the individuals with a level of *Arachnia* ≥ 0.768 have a higher chance of having gingival inflammation of 0.824 than the overall average of −0.00285. In contrast, 23.0% of the individuals with a level of *Arachnia* < 0.768 have a lower chance of having gingival inflammation of −0.249 than the overall average of 0.00285 (Figure 1). It can also be interpreted, in a clinical sense, that the administration of *Arachnia* to make it beyond the level of 0.768 is beneficial to prevent gingival inflammation. The CV error plot to show the underlying CV process can be found in the Supplementary Materials, Figure S2.

### 3.4. Data Mining: Random Forest

In this module, users can conduct microbiome data mining using random forest [16] for both classification and regression problems through CV for an optimal number of randomly selected taxa. First, users need to select an output (e.g., health or disease status) variable. Then, users select a data format among CLR (default), rarefied count, proportion and arcsine root. Then, users select covariates (e.g., age and sex) for the covariate-adjusted analysis or not for the unadjusted analysis. Then, users select a loss function, Gini impurity (default) for classification and mean squared error (default) for regression. Then, users select (i) 5- (default) or 10-fold CV, (ii) the number of bagged trees to be aggregated (default is 5000), and (iii) the maximum number of taxa to be displayed in later variable importance and partial dependence plots (default is 20). Finally, users select the taxonomic ranks to be surveyed ”from phylum to genus” (default), which is for 16S data [40,41], or ”from phylum to species”, which is for shotgun metagenomic data [47]. MiTree reports the main results using a variable importance plot that ranks the influence of microbial taxa on prediction (i.e., decrease in Gini impurity for classification and decrease in mean squared error for regression) and a partial dependence plot that shows the (possibly discrete or irregular) patterns of the relationships between taxonomic abundance and the output (health or disease status) values. MiTree reports supplemental results using a CV error plot to show the underlying CV process to search for an optimal tuning (the number of randomly selected taxa to create a tree) parameter value and an out-of-bag (OOB) error plot to show if the number of bagged trees was large enough for a sufficient convergence of the OOB error for the number of bagged trees to be aggregated.

**Figure 2:**
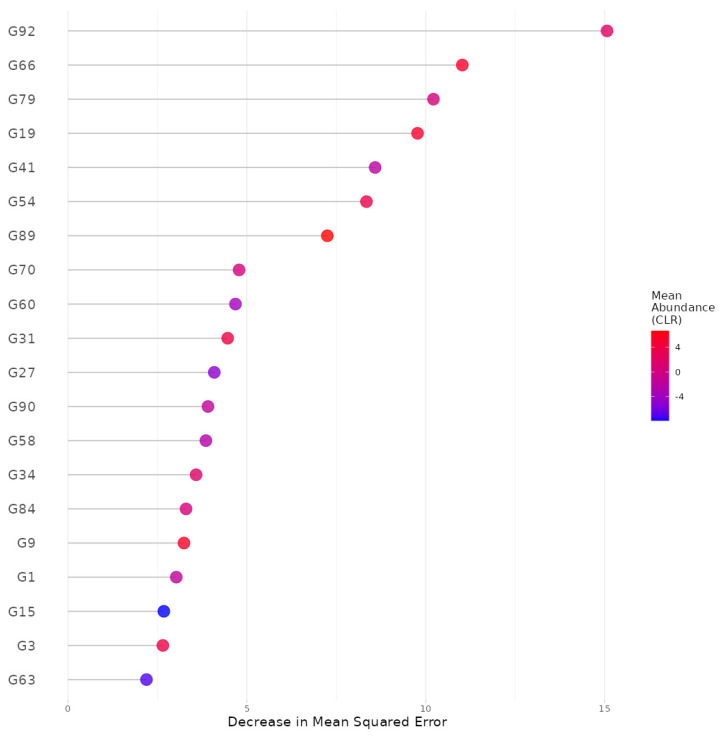
The variable importance plot that ranks the influence of genera on prediction of gingival inflammation resulting from random forest. G92: *Arachnia*, G66: *Neisseria*, G79: *Saccharibacteria_(TM7)_[G-3]*, G19: *Leptotrichia*, G41: *Shuttleworthia*, G54: *Parvimonas*, G89: *Streptococcus*, G70: *Pseudomonas*, G60: *Peptostreptococcaceae_[XI][G-2]*, G31: *Rothia*, G27: *Bifidobacterium*, G90: *Lactobacillus*, G58: *Mogibacterium*, G34: *Fretibacterium*, G84: *Megasphaera*, G9: *Capnocytophaga*, G1: *Gracilibacteria_(GN02)_[G-2]*, G15: *Eggerthia*, G3: *Bergeyella*, G63: *Desulfovibrio*.

**Figure 3:**
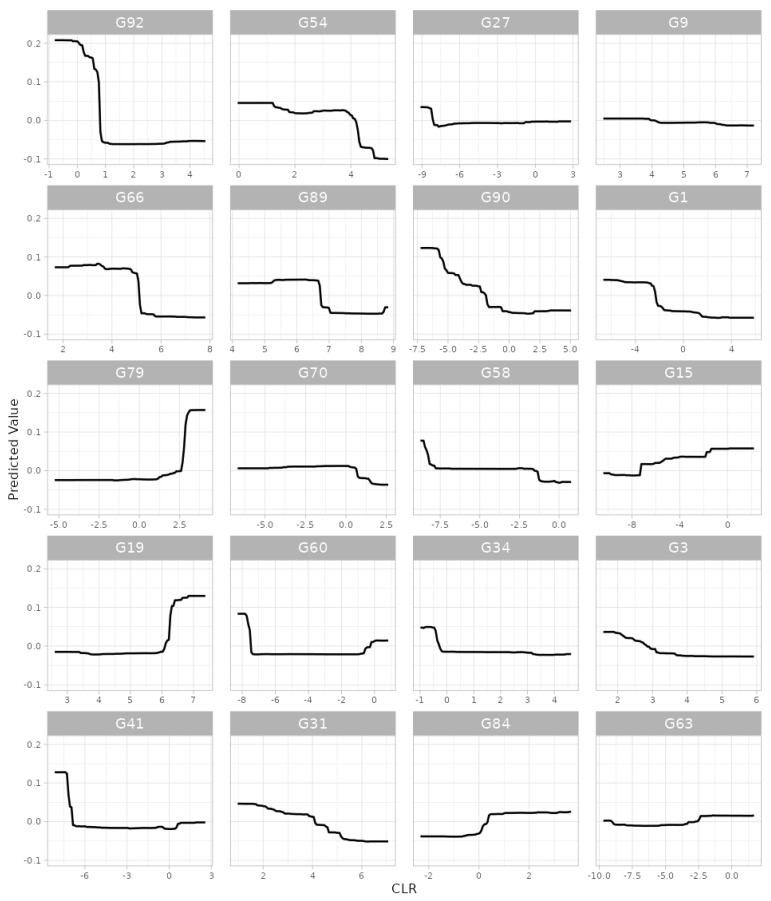
The partial dependence plot that shows the patterns of the relationships between taxonomic abundance and gingival inflammation resulting from random forest. G92: *Arachnia*, G66: *Neisseria*, G79: *Saccharibacteria_(TM7)_[G-3]*, G19: *Leptotrichia*, G41: *Shuttleworthia*, G54: *Parvimonas*, G89: *Streptococcus*, G70: *Pseudomonas*, G60: *Peptostreptococcaceae_[XI][G-2]*, G31: *Rothia*, G27: *Bifidobacterium*, G90: *Lactobacillus*, G58: *Mogibacterium*, G34: *Fretibacterium*, G84: *Megasphaera*, G9: *Capnocytophaga*, G1: *Gracilibacteria_(GN02)_[G-2]*, G15: *Eggerthia*, G3: *Bergeyella*, G63: *Desulfovibrio*.

Example: We selected gingival inflammation as the output variable and CLR [44] as the data format. Then, for the covariate-adjusted analysis we selected age, sex and the frequency of brushing teeth as covariates. Then, we used all the default settings for the rest of the widgets. In summary, based on the results, we found, at the genus level, that *Arachnia*, *Neisseria*, *Saccharibacteria_(TM7)_[G-3]*, *Leptotrichia*, *Shuttleworthia*, *Parvimonas*, *Streptococcus*, *Pseudomonas*, *Peptostreptococcaceae _[XI][G-2]*, *Rothia*, *Bifidobacterium*, *Lactobacillus*, *Mogibacterium*, *Fretibacterium*, *Megasphaera*, *Capnocytophaga*, *Gracilibacteria_(GN02)_[G-2]*, *Eggerthia*, *Bergeyella* and *Desulfovibrio* were the top 20 important predictors for gingival inflammation (Figure 2). We also found highly discrete and irregular patterns of the relationships between their taxonomic abundance and gingival inflammation (Figure 3). For example, as the abundance of *Arachnia* increases at the beginning, the occurrence of gingival inflammation is less likely, although as the abundance of *Arachnia* increases far beyond, it is not influential in gingival inflammation (Figure 3). It is also interpreted, in a clinical sense, that the administration of *Arachnia* is beneficial for individuals that are deplete in *Arachnia* to prevent gingival inflammation, but it is not helpful for the individuals that are already rich in *Arachnia*. The CV error plot to show the underlying CV process (Figure S3) and the OOB error plot to show a sufficient convergence of the OOB error for the number of bagged trees to be aggregated can also be found in the Supplementary Materials, Figure S4.

### 3.5. Data Mining: Gradient Boosting

In this module, users can conduct microbiome data mining using gradient boosting [17] through the software package, XGBoost [18], for both classification and regression problems. First, users need to select an output (e.g., health or disease status) variable. Then, users select a data format among CLR (default), rarefied count, proportion, and arcsine root. Then, users select covariates (e.g., age and sex) for the covariate-adjusted analysis or not for the unadjusted analysis. Then, users select a loss function, cross entropy (default), area under the curve (AUC) or misclassification error rate for classification and mean squared error (default) for regression. Then, users select (i) 5- (default) or 10-fold CV, (ii) the maximum number of iterations (updates) in the boosting process (the default is 5000), (iii) the learning rate (the default is 0.005), (iv) the use of regularization on leaves (yes (default) or no), and (v) the maximum number of taxa to be displayed in later variable importance and partial dependence plots (the default is 20). Finally, users select the taxonomic ranks to be surveyed ”from phylum to genus” (default), which is for the 16S data [40,41], or ”from phylum to species”, which is for the shotgun metagenomic data [47]. MiTree reports the main results using (1) a variable importance plot that ranks of influence of microbial taxa on prediction based on Shapley additive explanation (SHAP) values [48], and (2) a partial dependence plot that shows the (possibly discrete or irregular) patterns of the relationships between taxonomic abundance and the output (health or disease status) values. MiTree reports supplemental results using a CV error plot to describe the underlying CV process to search for an optimal tuning (the number of iterations (updates) in the boosting process) parameter value. 

**Figure 4:**
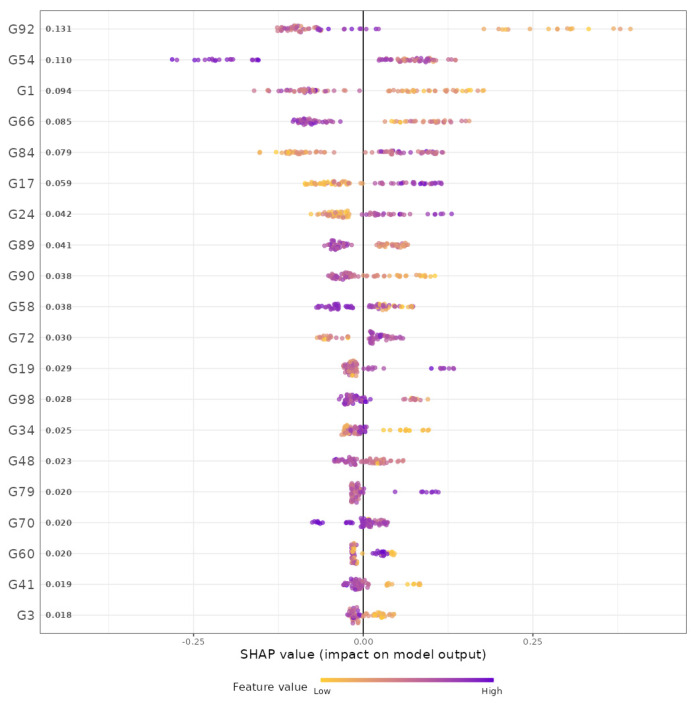
The variable importance plot that ranks the influence of genera on prediction of gingival inflammation resulting from gradient boosting. The purple (yellow) units on the left side of the plot implies that the taxon is enriched (deplete), while the purple (yellow) units on the right side of the plot implies that the taxon is enriched (deplete). G92: *Arachnia*, G54: *Parvimonas*, G1: *Gracilibacteria_(GN02)_[G-2]*, G66: *Neisseria*, G84: *Megasphaera*, G17: *Bulleidia*, G24: *Cryptobacterium*, G89: *Streptococcus*, G90: *Lactobacillus*, G58: *Mogibacterium*, G72: *Aggregatibacter*, G19: *Leptotrichia*, G98: *Veillonellaceae_[G-1]*, G34: *Fretibacterium*, G48: *Ruminococcaceae_[G-2]*, G79: *Saccharibacteria_(TM7)_[G-3]*, G70: *Pseudomonas*, G60: *Peptostreptococcaceae_[XI][G-2]*, G41: *Shuttleworthia*, G3: *Bergeyella*.

**Figure 5:**
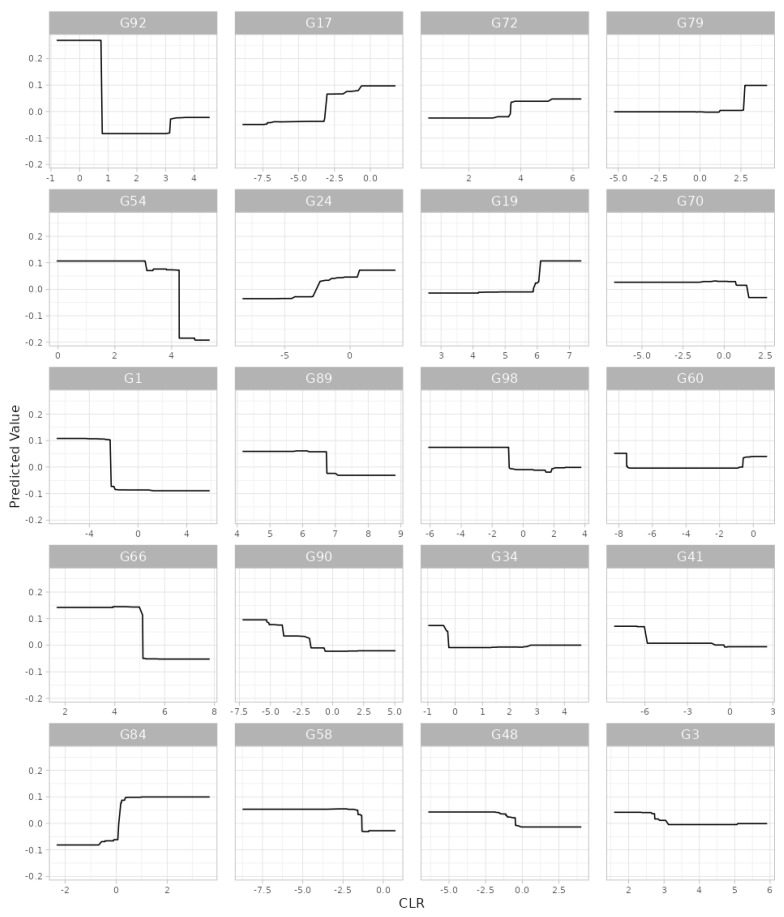
The partial dependence plot that shows the patterns of the relationships between taxonomic abundance and gingival inflammation resulting from gradient boosting. G92: *Arachnia*, G54: *Parvimonas*, G1: *Gracilibacteria_(GN02)_[G-2]*, G66: *Neisseria*, G84: *Megasphaera*, G17: *Bulleidia*, G24: *Cryptobacterium*, G89: *Streptococcus*, G90: *Lactobacillus*, G58: *Mogibacterium*, G72: *Aggregatibacter*, G19: *Leptotrichia*, G98: *Veillonellaceae_[G-1]*, G34: *Fretibacterium*, G48: *Ruminococcaceae_[G-2]*, G79: *Saccharibacteria_(TM7)_[G-3]*, G70: *Pseudomonas*, G60: *Peptostreptococcaceae_[XI][G-2]*, G41: *Shuttleworthia*, G3: *Bergeyella*.

Example: We selected gingival inflammation as the output variable and CLR [44] as the data format. Then, for the covariate-adjusted analysis, we selected age, sex and the frequency of brushing teeth as covariates. Then, we used all the default settings for the rest of the widgets. In summary, based on the results, we found, at the genus level, that *Arachnia*, *Parvimonas*, *Gracilibacteria_(GN02)_[G-2]*, *Neisseria*, *Megasphaera*, *Bulleidia*, *Cryptobacterium*, *Streptococcus*, *Mogibacterium*, *Aggregatibacter*, *Leptotrichia*, *Veillonellaceae_[G-1]*, *Fretibacterium*, *Ruminococcaceae_[G-2]*, *Saccharibacteria_(TM7)_[G-3]*, *Pseudomonas*, *Peptostreptococcaceae_[XI][G-2]*, *Shuttleworthia* and *Bergeyella* were the top 20 important predictors for gingival inflammation (Figure 4). We also found highly irregular patterns of the relationships between their taxonomic abundance and gingival inflammation (Figure 5). For example, as the abundance of *Arachnia* increases at the beginning, the occurrence of gingival inflammation is less likely, although as the abundance of *Arachnia* increases far beyond, it is not influential in gingival inflammation (Figure 5). It is also interpreted, in a clinical sense, that the administration of *Arachnia* is beneficial for individuals that are deplete in *Arachnia* to prevent gingival inflammation, but it is not helpful for the individuals that are already rich in *Arachnia*. The CV error plot to show the underlying CV process can be found in the Supplementary Materials, Figure S5.

Example (Ask ChatGPT): We asked ChatGPT a query, i.e., “Tell me about the roles of *Arachnia* on gingival inflammation”, while selecting “genus” as a taxonomic rank of interest and “*Arachnia*” as a microbial taxon of interest to search for prior knowledge on its roles of gingival inflammation. Then, ChatGPT answered and reported the search results from Google Scholar and PubMed as in the Supplementary Materials, Figure S6. 

## 4. Conclusions

The field of human microbiome studies is rapidly growing. Investigators are actively seeking new ways to diagnose, treat and prevent human diseases through the human microbiome. A promising approach has been to use machine learning due to the high complexity of microbiome data. However, many of the current machine learning algorithms are difficult to understand and interpret. Investigators are also curious about many different aspects, such as which microbial taxa are culprits or fellow-travelers and how they are related to human diseases. Many investigators in different disciplines (e.g., clinicians, public health practitioners and biologists) are also not highly skilled at computer programming.

In this paper, we introduced MiTree, a unified web cloud analytic platform for user-friendly and interpretable microbiome data mining. MiTree incorporates tree-based learning methods, i.e., decision tree [15], random forest [16] and gradient boosting [17,18], which are well understood and suited to highly complex microbiome data. MiTree is also unique in microbiome-based disease prediction with the facilities of a covariate-adjusted analysis for both classification and regression problems. Furthermore, MiTree is easy to understand and interpret with clear and accurate visualizations. To summarize, MiTree should be attractive as a user-friendly and interpretable microbiome data mining tool for many researchers in various fields (e.g., biology, public health and medicine), while providing new insights into the microbiome-based diagnostics, treatment and prevention.

We demonstrated the use of MiTree with diagnostic and clinical interpretations through an example oral microbiome study to predict gingival inflammation (output) using microbial taxa (inputs) [39]. Since we uploaded the processed data as example data (see *Data Input: Example Data* on our web server: http://mitree.micloud.kr, 10 October 2023), users can also easily follow our analyses, results and interpretations. 

Lastly, as a great statistician, John Wilder Tukey, described “Today, the ‘software’ comprising the carefully planned interpretive routines, compilers, and other aspects of automative programming are at least as important to the modern electronic calculators as its ‘hardware’ of tubes, transistors, wires, tapes and the like” in [49], we believe that the ”software” is important and it makes the use of existing statistical methods painless. However, it does not mean that the software developers can take all the credit for all the underlying protocols and methods. Thus, in MiTree, we list all related prior studies as references on its user interfaces, which we also did for our prior web cloud platforms [26,27,28,29], but unfortunately, it is difficult to find any other (either web-based or command-line-based) software to do so. 

However, MiTree can apply to cross-sectional studies with a binary or continuous output variable, but in reality, there are various study designs (e.g., cross-sectional, family-based, repeated measures, paired, block and follow-up study designs), output variable types (e.g., binary, continuous, time-to-event and multi-category [50] outputs) and microbiome data (e.g., pathways and strain-level markers [51]). Therefore, further extensions are needed for more comprehensive microbiome data mining. 

## Figures and Tables

**Table 1:** A comparison across the tree-based methods, i.e., decision tree, random forest, gradient boosting. * We suggest that random forest or gradient boosting is used as a main analytic method because of their high accuracy in prediction.

Criteria	Decision Tree	Random Forest *	Gradient Boosting *
Interpretability	Very easy	Easy	Easy
Prediction accuracy	Moderate	Very accurate	Very accurate
Computational speed	Very fast	Moderate	Slow

**Table 2:** A comparison of MiTree to our prior web cloud platforms: MiCloud, MiPair, MiSurv and MiMed.

Platform	Main Facility	Output Variable	Covariate-Adjustment	Study Design
MiCloud	Association testing	Binary/continuous	Yes	Cross-sectional, Family-based, repeated measures
MiPair	Paired analysis	Continuous	No	Paired/Block
MiSurv	Survival analysis	Time-to-event	Yes	Follow-up
MiMed	Mediation analysis	Binary/continuous	Yes	Cross-sectional
MiTree	Prediction modeling	Binary/continuous	Yes	Cross-sectional

## Data Availability

Data are contained within the article.

## Supplementary Materials

The following supporting information can be downloaded at: https://www.mdpi.com/article/10.3390/microorganisms11112816/s1. Figure S1. The screenshot of MiTree after quality controls. Figure S2. The underlying CV process for cost-complexity pruning in decision tree to search for an optimal tuning parameter (the number of leaves or complexity parameter) value. Figure S3. The underlying CV process for random forest to search for an optimal tuning parameter (the number of randomly selected taxa to create a tree) value. Figure S4. The OOB error plot from random forest to show a sufficient convergence of the OOB error for the number of bagged trees to be aggregated (5000). Figure S5. The underlying CV process for gradient boosting to search for an optimal tuning parameter (the number of iterations (updates) in the boosting process) value. Figure S6. The screenshot of the Ask ChatGPT plugin.
