# Supplementary material for: MiTree: A Unified Web Cloud Analytic Platform for User-Friendly and Interpretable Microbiome Data Mining Using Tree-Based Methods
Source: Microorganisms. 2023 Nov 20;11(11):2816. doi: 10.3390/microorganisms11112816 (PMC10672986; doi:10.3390/microorganisms11112816)

Supplemental Material

Figure S1. The screenshot of MiTree after quality controls.

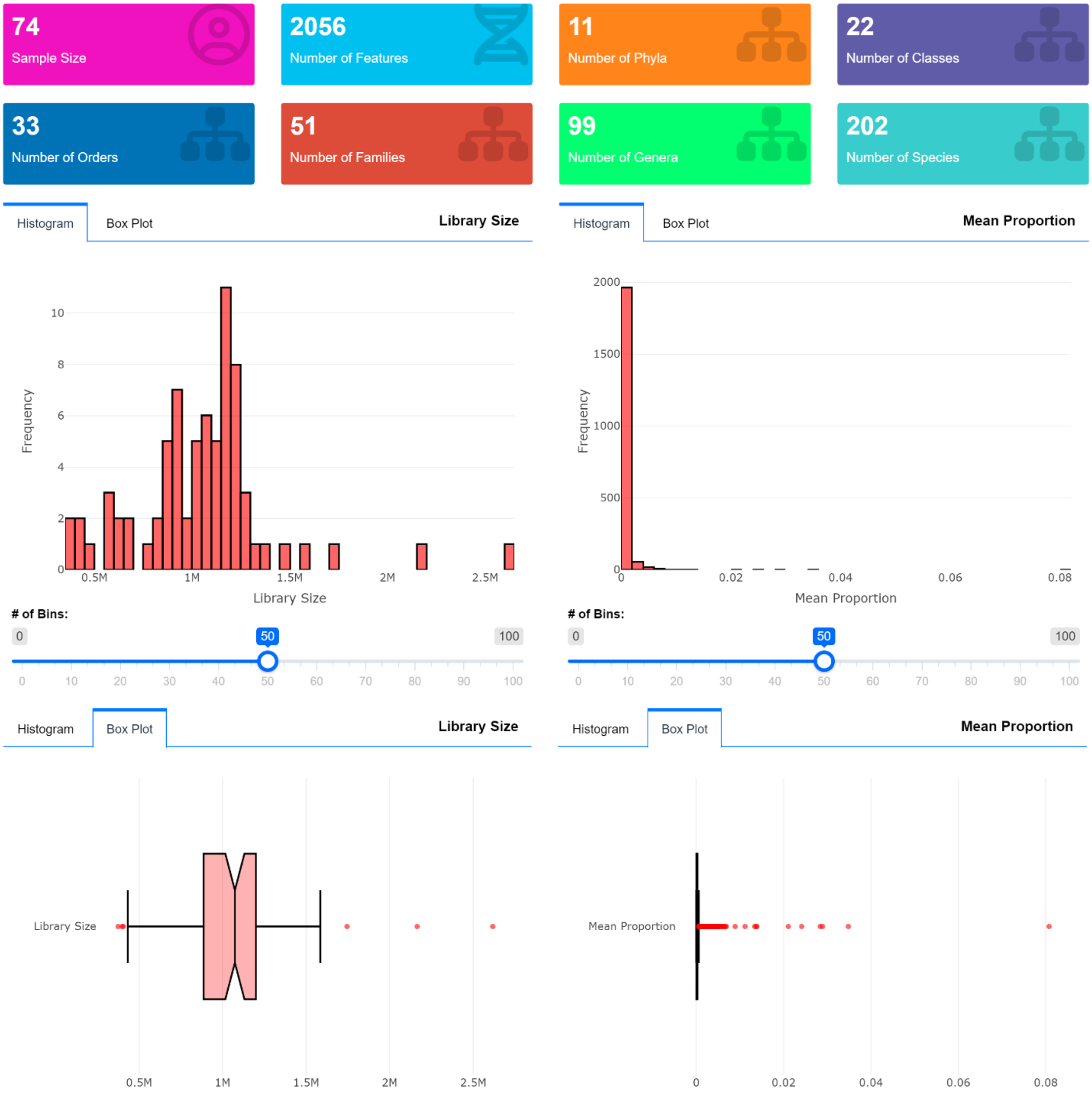

Figure S2. The underlying CV process for cost-complexity pruning in decision tree to search for an optimal tuning parameter (the number of leaves or complexity parameter) value.

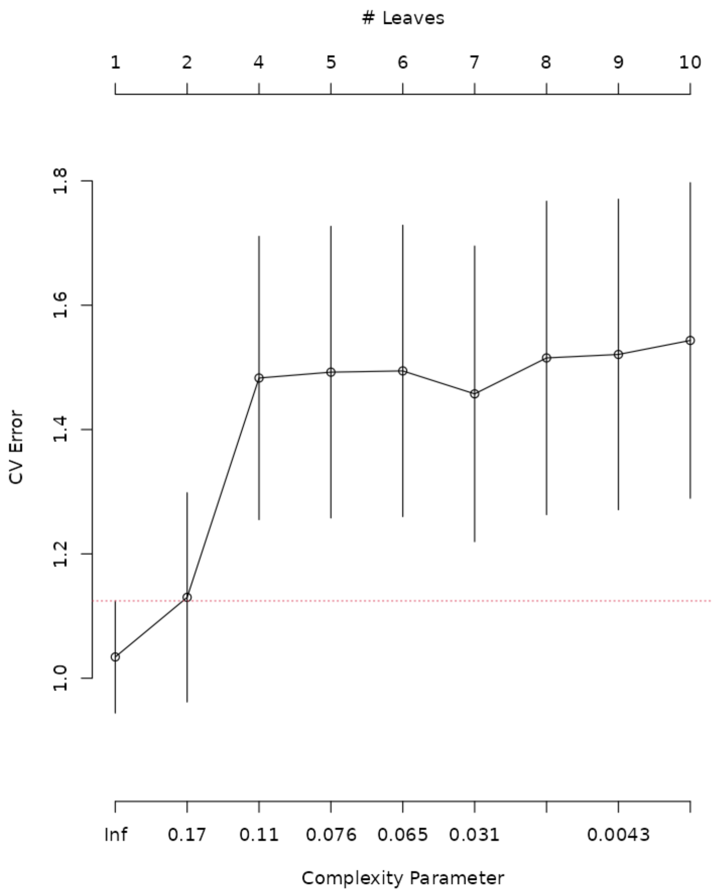

Figure S3. The underlying CV process for random forest to search for an optimal tuning (the number of randomly selected taxa to create a tree) parameter value.

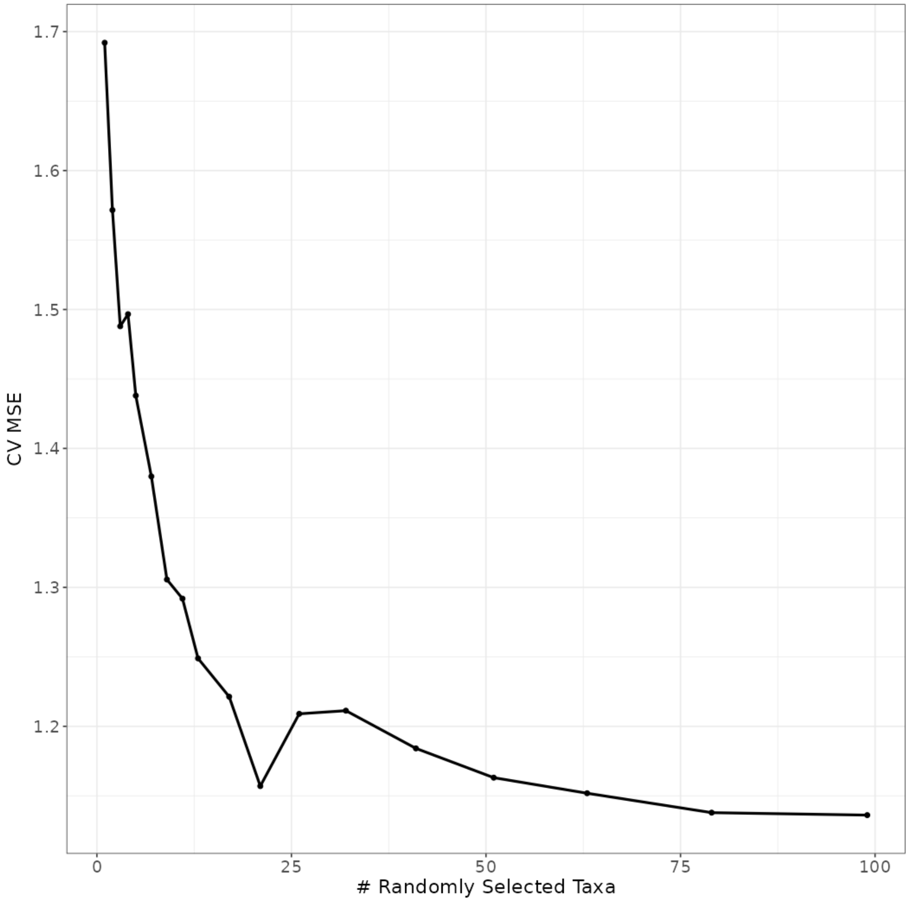

Figure S4. The OOB error plot from random forest to show a sufficient convergence of the OOB error for the number of bagged trees to be aggregated (5,000).

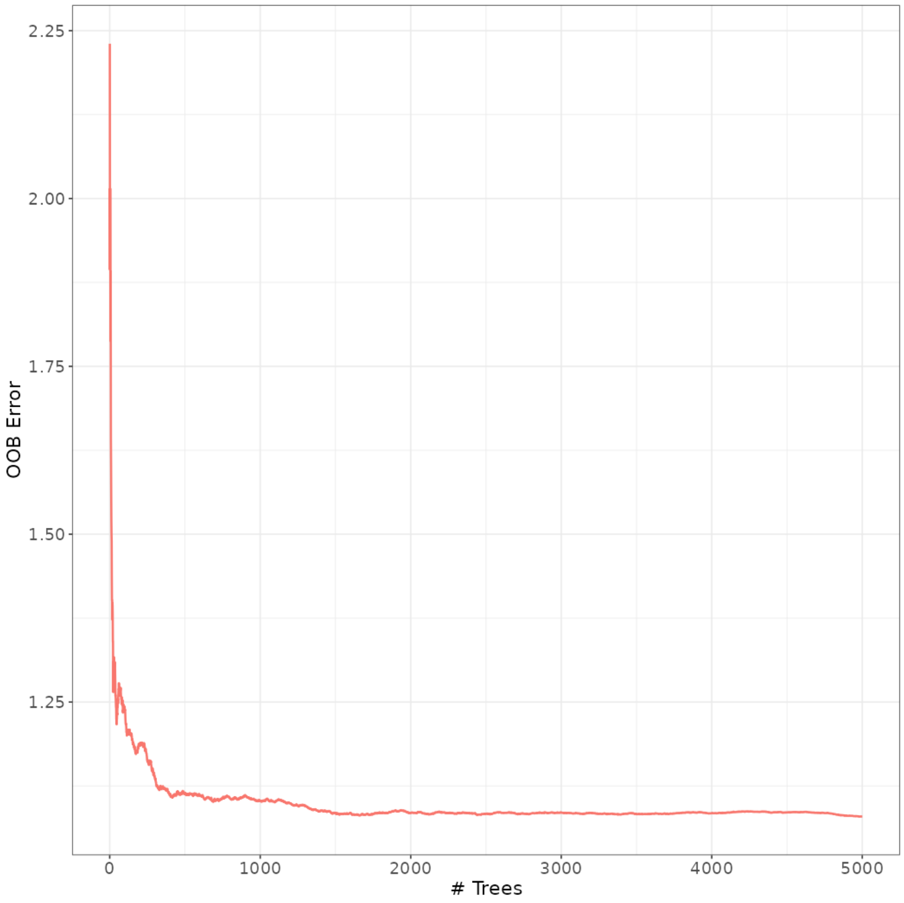

Figure S5. The underlying CV process for gradient boosting to search for an optimal tuning (the number of iterations (updates) in the boosting process) parameter value.

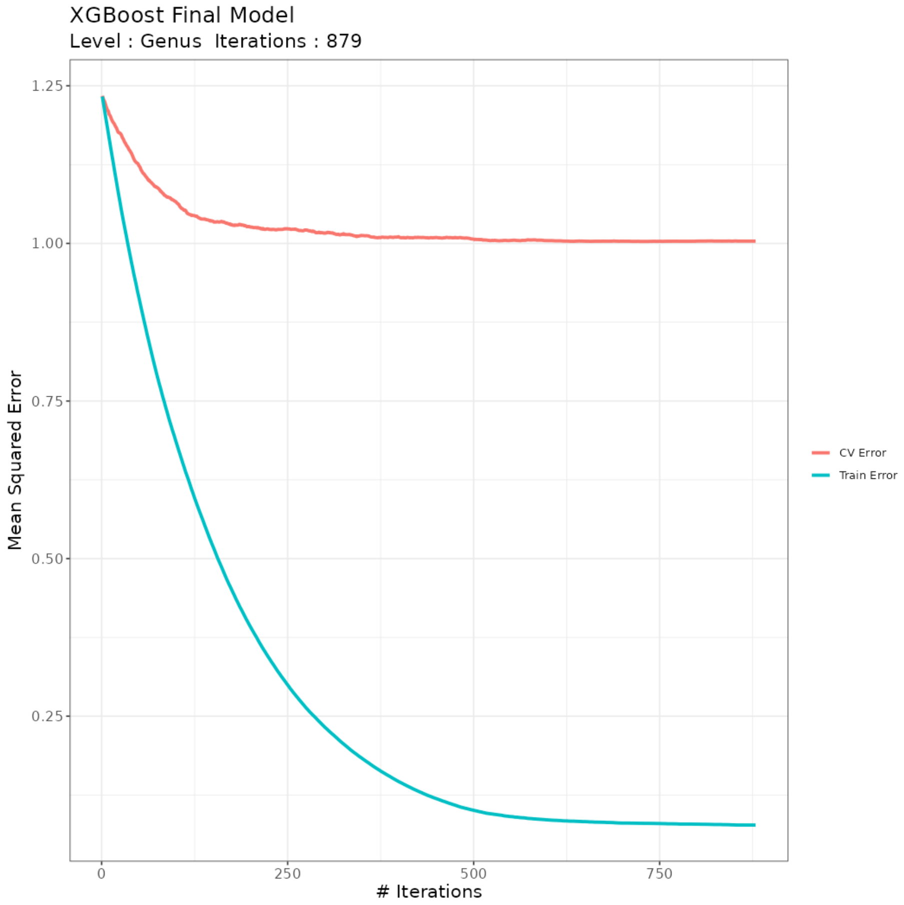

Figure S6. The screenshot of the Ask ChatGPT plugin.

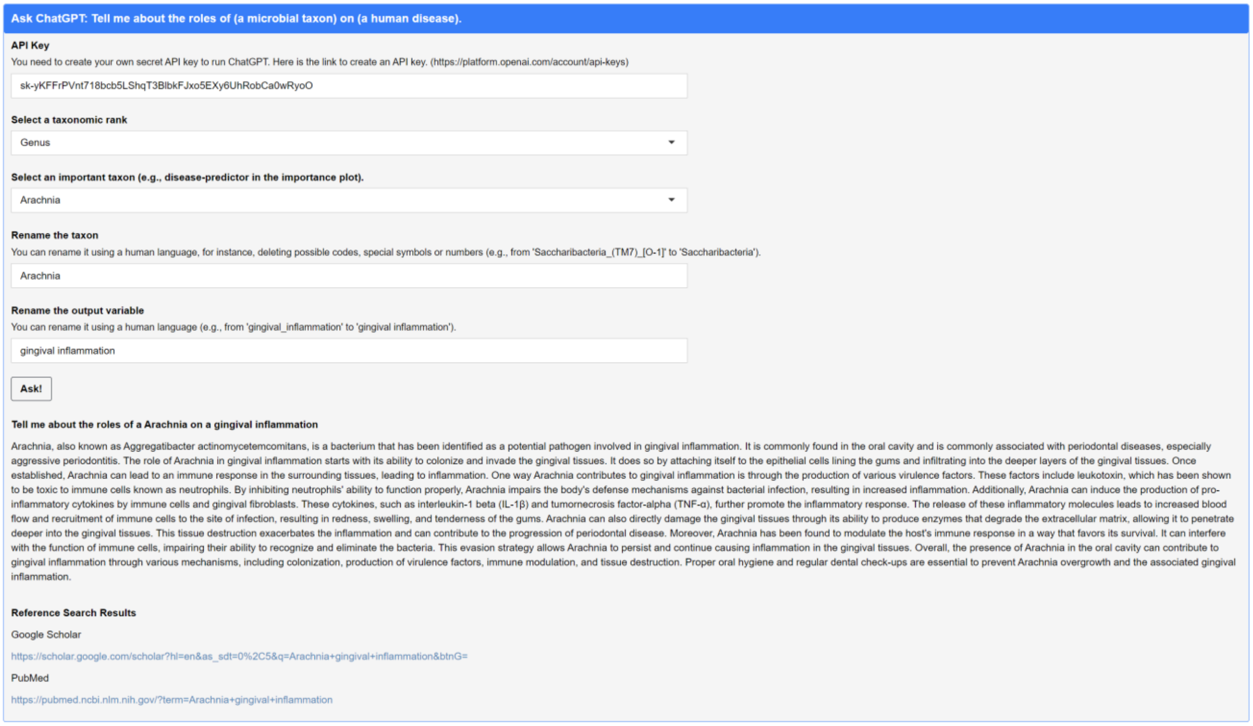

Supplement: Supplementary file 1 [file microorganisms-11-02816-s001.zip › microorganisms-2685409-supplementary.pdf]
